# Polarized hard X-ray photoemission system with micro-positioning technique for probing ground-state symmetry of strongly correlated materials

**DOI:** 10.1107/S1600577516003003

**Published:** 2016-04-01

**Authors:** Hidenori Fujiwara, Sho Naimen, Atsushi Higashiya, Yuina Kanai, Hiroshi Yomosa, Kohei Yamagami, Takayuki Kiss, Toshiharu Kadono, Shin Imada, Atsushi Yamasaki, Kouichi Takase, Shintaro Otsuka, Tomohiro Shimizu, Shoso Shingubara, Shigemasa Suga, Makina Yabashi, Kenji Tamasaku, Tetsuya Ishikawa, Akira Sekiyama

**Affiliations:** a Division of Materials Physics, Graduate School of Engineering Science, Osaka University, Toyonaka, Osaka 560-8531, Japan; b SPring-8/RIKEN, Sayo, Hyogo 679-5148, Japan; c Faculty of Science and Engineering, Setsunan University, Neyagawa, Osaka 572-8508, Japan; d Department of Physical Science, Ritsumeikan University, Kusatsu, Shiga 525-8577, Japan; e Faculty of Science and Engineering, Konan University, Kobe 658-8501, Japan; f Department of Physics, College of Science and Technology, Nihon University, Chiyoda, Tokyo 101-0062, Japan; g Graduate School of Science and Technology, Kansai University, Suita, Osaka 564-8680, Japan; h Institute of Scientific and Industrial Research, Osaka University, Ibaraki, Osaka 567-0047, Japan

**Keywords:** hard X-ray photoemission, linear dichroism, strongly correlated electron systems, phase retarder, micro-focused X-rays, sample monitoring system, low-temperature double-axis manipulator

## Abstract

An angle-resolved linearly polarized hard X-ray photoemission spectroscopy (HAXPES) system has been developed to study the ground-state symmetry of strongly correlated materials. The linear polarization of the incoming X-ray beam is switched by a transmission-type phase retarder composed of two diamond (100) crystals. The best value of the degree of linear polarization was found to be −0.96, containing a vertical polarization component of 98%. A newly developed low-temperature two-axis manipulator enables easy polar and azimuthal rotations to select the detection direction of photoelectrons. The lowest temperature achieved was 9 K, offering the chance to access the ground state even for strongly correlated electron systems in cubic symmetry. A co-axial sample monitoring system with long-working-distance microscope enables the same region on the sample surface to be measured before and after rotation. Combining this sample monitoring system with a micro-focused X-ray beam by means of an ellipsoidal Kirkpatrick–Baez mirror (25 µm × 25 µm FWHM), polarized valence-band HAXPES has been performed on NiO for voltage application as resistive random access memory to demonstrate the micro-positioning technique and polarization switching.

## Abstract 2

A linearly polarized hard X-ray photoemission system has been developed for studying the ground-state symmetry of strongly correlated materials.

## 1. Introduction   

Strongly correlated electron systems show a variety of anomalous phenomena such as high-*T*_c_ superconductivity and metal–insulator transitions, attracting much attention in solid state physics. In the case of transition-metal 3*d*-electron systems, the ground-state symmetry can easily be determined due to the crystal-field splitting of eV order. However, the situation is different in the case of rare-earth 4*f*-electron systems in which the crystal-field splitting is usually of meV order. Therefore, the determination of the ground-state symmetry for the 4*f*-electron systems is still a non-trivial issue, although it is crucial for discussing the origin of intriguing phenomena such as quantum criticality, heavy-Fermion superconductivity and non-Fermi liquid behavior. For this purpose, the linear polarization dependence of soft X-ray absorption spectra (XAS) has been well established as a local probe to detect the symmetry of the electronic structure due to excitonic excitation forming a core hole in the final states with dipole selection rules. This technique is powerful for Ce compounds with tetragonal or orthorhombic crystal structures (Hansmann *et al.*, 2008 ▸; Willers *et al.*, 2009 ▸, 2010 ▸, 2012 ▸, 2015 ▸; Strigari *et al.*, 2012 ▸, 2013 ▸), but does not work for a high symmetric cubic system because the selection rule is only sensitive to cases where the electric field vector of the excitation photon is parallel or perpendicular to the anisotropic crystal axis. This fundamental limitation is, however, overcome by the recent development of angle-resolved ‘linealy polarized’ hard X-ray photoemission spectroscopy (HAXPES) (Mori *et al.*, 2014 ▸), in which dipole selection rules work equally well as polarized XAS. Moreover, we have another controllable probing parameter, *i.e.* the photoelectron-detection direction, enabling the ground-state symmetry to be probed even for cubic systems (Kanai *et al.*, 2015 ▸). This type of polarized HAXPES is a brand new spectroscopy for discussing the ground-state symmetry in contrast to the so far reported polarized HAXPES technique which is mainly used to study the electronic structures utilizing the angular-momentum dependence of photoelectron angular distributions due to the atomic photo-ionization cross section (Sekiyama *et al.*, 2010 ▸, 2013 ▸; Nakatsu *et al.*, 2011 ▸; Weinen *et al.*, 2015 ▸) or magnetic circular dichroism (Ueda *et al.*, 2008 ▸; Ouardi *et al.*, 2011 ▸; Kozina *et al.*, 2011 ▸).

In this paper we report on the performance of a HAXPES system with polarization switching potential at SPring-8 BL19LXU. Double phase retarders can switch the polarization of the incoming photon from horizontal to vertical polarization with the degree of linear polarization *P*_L_ = −0.96. A two-axis manipulator with lowest available temperature of 9 K has been newly developed to easily select the photoelectron-detection direction in the ground state of strongly correlated electron systems. Moreover, a sample monitoring system with co-axial long-working-distance microscope enables one to keep measuring the same sample region after rotation. Combined with a micro-focused X-ray beam of size 25 µm × 25 µm (FWHM), we further demonstrate the linear polarization dependence of the valence-band HAXPES spectra for voltage-applied NiO surrounded by 200 µm × 200 µm Pt markers to test the polarization switching and the micro-positioning technique.

## 2. HAXPES system with linear polarization switching   

### 2.1. Beamline setup and double-crystal phase retarder   

**Figure 1:**
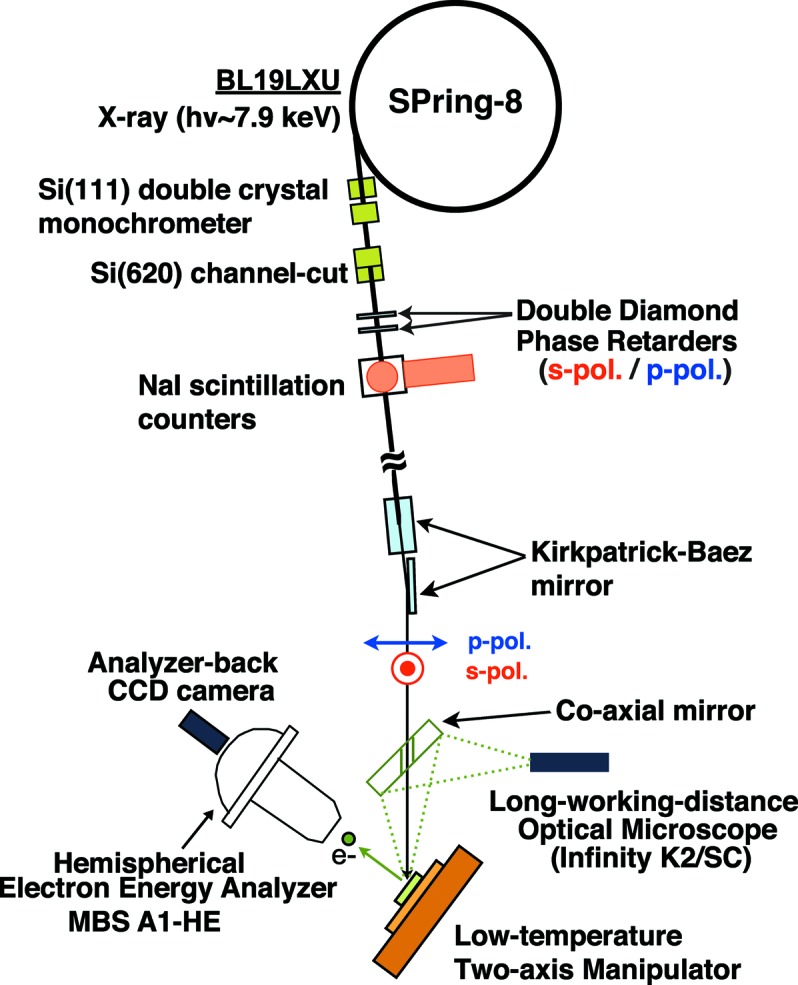
Overview of the experimental geometry (top view) for polarized HAXPES at BL19LXU in SPring-8.

**Figure 2:**
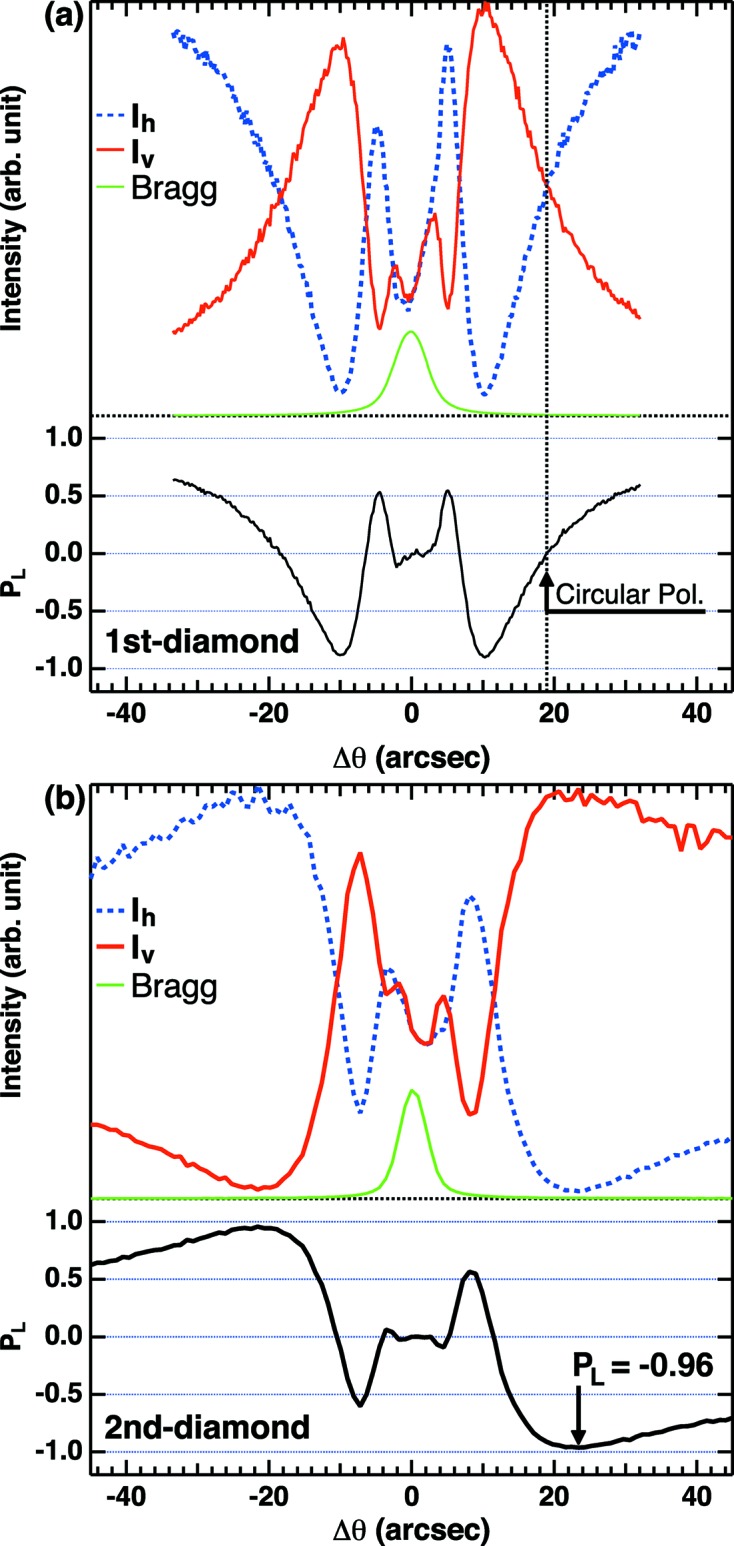
Offset angle dependence of the photon intensities relative to the diamond (220) Bragg reflections for the first (*a*) and second (*b*) diamond. The evaluated degree of linear polarization *P*_L_ is also plotted at the bottom of (*a*) and (*b*).

Polarization-dependent HAXPES was performed at BL19LXU at SPring-8 using an MBS A1-HE hemispherical photoelectron spectrometer which is inclined at 60° from the incident X-ray beam. Fig. 1 ▸ shows a schematic diagram of the typical setting of the beamline optics for polarized HAXPES. Linearly polarized light is delivered from an in-vacuum 27 m-long 780 period undulator (Kitamura *et al.*, 2001 ▸; Yabashi *et al.*, 2001*a* ▸,*b* ▸). The photon energy is set to ∼7.9 keV by the Si(111) double-crystal monochrometer and further monochromated by the Si(620) channel-cut crystal. The transmission-type phase retarder made of two single-crystalline (100) diamonds is placed downstream of the channel-cut crystal to switch the polarization of the X-ray beam (Hirano *et al.*, 1991 ▸, 1993 ▸; Hirano & Maruyama, 1997 ▸; Giles *et al.*, 1994 ▸). To compensate for the phase-shift inhomogeneity due to the angular divergence of the incoming X-ray beam, we use the double-phase-plate configuration (Okitsu *et al.*, 2001 ▸), giving the (220) Bragg reflection with Laue geometry, whose scattering plane is inclined at 45° to the electric field vector of the incoming X-ray beam. The original horizontally polarized photons are transformed into circular polarization by the first diamond plate (Fig. 2*a* ▸), and then the offset angle of the second plate is scanned as shown in Fig. 2(*b*) ▸ to give a further phase shift for converting to vertical polarization (Scagnoli *et al.*, 2009 ▸). The degree of linear polarization is defined as
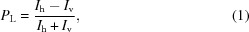
where 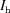 and 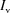 are intensities of the horizontal and vertical polarized photons, respectively. 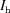 and 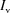 are evaluated using scattering photon intensities from polyimide film detected with NaI scintillation counters located downstream of the phase retarder. In our setup, the best *P*_L_ value is evaluated as −0.96 as shown in Fig. 2(*b*) ▸. This gives the fraction of vertical components of 98%, having sufficient quality to enable discussion of the linear polarization dependence of the photoemission spectra. Note that the electric field vector of the horizontal (vertical) polarized photons is parallel (perpendicular) to the photoelectron scattering plane, and thus defined as *p*-polarization (*s*-polarization). The thickness of each diamond plate is 0.25 mm, and the transmittance of the X-ray beam after the double-crystal phase retarder is ∼50%, which gives a much better throughput for the vertically polarized photons than that for a 0.7 mm-thick single phase-retarder setup with transmittance of ∼35% (Sekiyama *et al.*, 2010 ▸, 2013 ▸; Nakatsu *et al.*, 2011 ▸). Afterwards the X-ray beam is focused onto the sample within 25 µm × 25 µm (FWHM) using an ellipsoidal Kirkpatrick–Baez mirror as shown in Fig. 1 ▸.

### 2.2. Low-temperature two-axis manipulator   

**Figure 3:**
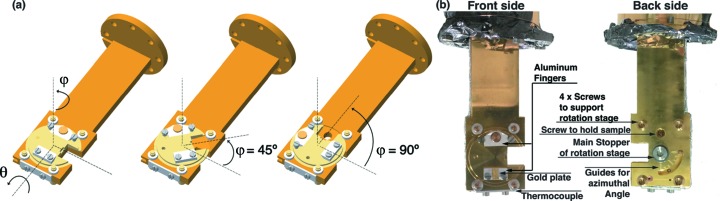
(*a*) Schematic view of our developed low-temperature two-axis manipulator with the definition of azimuthal angle φ and polar angle θ, and simulations for the azimuthal rotation with rotation angles of 45° (middle) and 90° (right). (*b*) Photographs of the manipulator taken from the front (left) and back (right).

We have developed a two-axis manipulator for polarized HAXPES to easily optimize the detection direction of photoelectrons. Fig. 3(*a*) ▸ shows schematic drawings of the manipulator. The body of the manipulator is made of oxygen-free copper with gold plating. The rotation feedthrough provides polar rotation (θ), and a rotation stage made of Be-Cu on top of the manipulator gives the azimuthal rotation (φ) over a 90° range. The bottom of the rotation stage is bowl-shaped to gain thermal contact, and is tightly fixed by a non-magnetic screw from the back. This screw is loosened during the azimuthal rotation, but four screws support the rotation stage to prevent it from falling during rotation, as shown in Fig. 3(*b*) ▸. By minimizing the volume of the manipulator, we have achieved a lowest temperature of 9 K using a closed-cycle He refrigerator, enabling cooling to 9 K in 1.5 h from 300 K. Although the azimuth stage loses thermal contact during rotation, one can immediately cool down the sample after re-tightening the rotation stage screw. The sample is mounted on the rotation stage by clamping between two aluminium fingers with a screw to hold the sample using a hexagonal wrench from the back, and the same wrench can be used to manipulate the rotation stage. To guide the rotation angle, there are seven indication marks every 15°.

**Figure 4:**
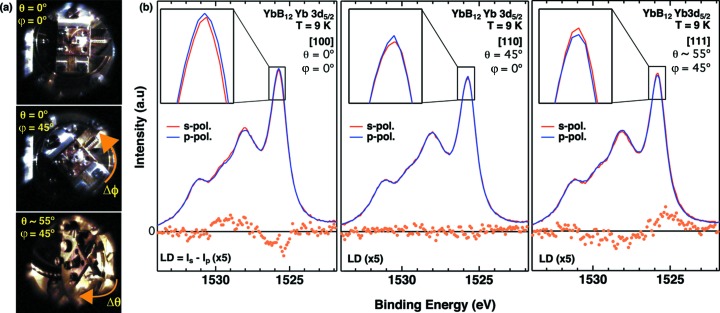
(*a*) CCD camera image of the manipulator (top) demonstrating the 45° rotation for the azimuthal axis (middle) and further polar rotation of ∼55°(bottom). (*b*) Linear polarization dependence of the Yb^3+^ 3*d*_5/2_ core-level spectra for cubic YbB_12_ by selecting the photoelectron-detection directions [100] (left), [110] (middle) and [111] (right). Insets are close-ups of the 1525.5 eV peak in the Yb^3+^ 3*d*_5/2_ spectra. The so-called Shirly-type background is subtracted as discussed in the literature (Kanai *et al.*, 2015 ▸).

The rotation angle is fixed more precisely by monitoring the sample with a charge-coupled device (CCD) camera, which is mounted on the back of the hemispherical analyzer. The camera image is captured and shown on computer screen using a program with a function to calculate the rotation angle with an accuracy of ±1°. Fig. 4(*a*) ▸ shows the rotation procedures of the two-axis manipulator recorded by this CCD camera with magnification of ∼3 achieved using a varifocal lens (Tamron: 13VM20100AS). The middle panel of Fig. 4(*a*) ▸ clearly shows the azimuthal rotation of 45°, and further polar rotation at ∼55° (bottom).

By selecting the photoelectron-detection direction with the two-axis manipulator, we have studied the polarized HAXPES for a cubic strongly correlated rare-earth compound YbB_12_ (Kanai *et al.*, 2015 ▸). Fig.  4(*b*) ▸ shows the Yb^3+^ 3*d*_5/2_ core-level spectra along the [100], [110] and [111] directions. Note that all three geometries have been set by the polar θ and azimuthal φ rotations without any sample transfer procedure. In the [100] emission geometry for (θ, φ) = (0°, 0°), the Yb^3+^ 3*d* core-level spectra show the polarization dependence especially at the peak at 1525.5 eV. As shown in the close-up of the peak top (inset), the *p*-polarized spectral weight is slightly stronger than the *s*-polarized spectral weight. This is highlighted by the linear dichroism (LD) in the core-level spectra (bottom) defined as 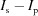, where 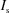 (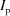) denotes the intensity of *s*-polarized (*p*-polarized) spectra. In the [100] emission geometry the LD spectrum changes sign near 1527 eV. This difference is noticeably suppressed in the [110] emission geometry by rotating the polar angle θ by 45°, and again shows up with the [111] emission geometry after rotation for both θ and φ axis of the (θ, φ) = (∼55°, 45°) setting in Fig. 4(*a*) ▸. It is most interesting that the sign of LD for the [111] emission geometry is the reverse of that for the [100] emission, revealing the ground-state symmetry of the Yb^3+^ 4*f* states due to the Coulomb and exchange interactions between the Yb 3*d* core and 4*f* holes (Mori *et al.*, 2014 ▸; Kanai *et al.*, 2015 ▸). Thus we stress that the selection of the photoelectron-detection direction is crucial for probing the ground-state symmetry for various strongly correlated electron systems.

### 2.3. Co-axial monitoring system with long-working-distance microscope   

**Figure 5:**
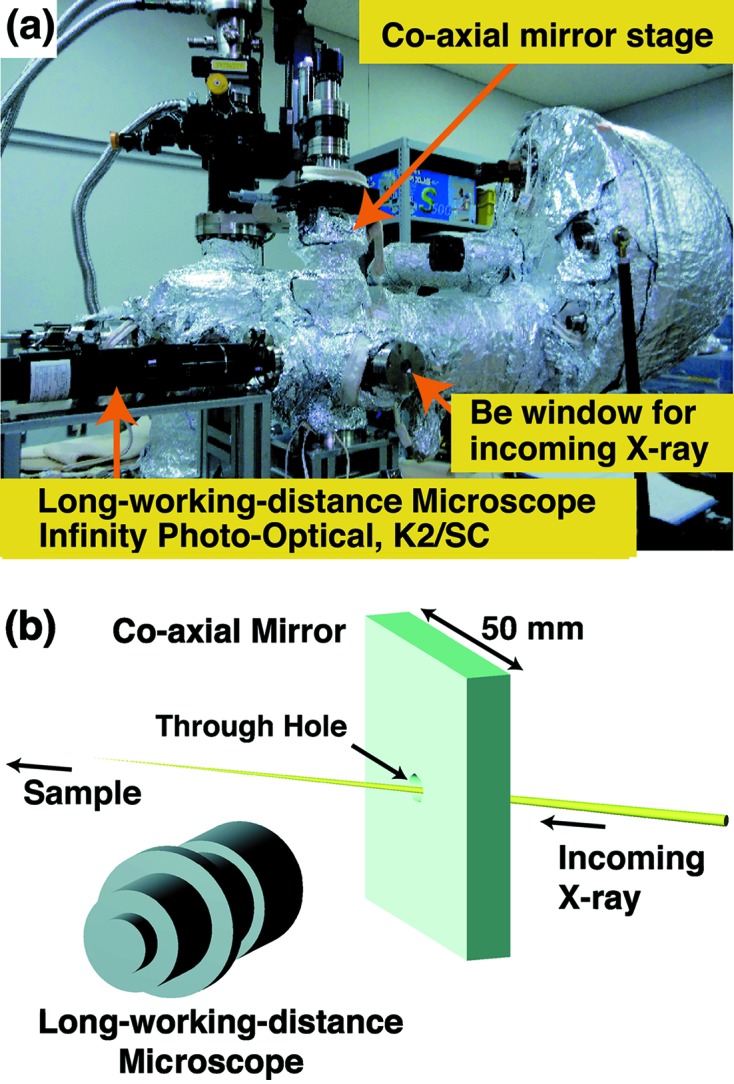
(*a*) Photograph of the co-axial sample monitoring system combining the long-working-distance microscope with co-axial mirror, and (*b*) schematic view. The top view of the optical geometry is shown in Fig. 1 ▸.

The two-axis rotation is very powerful for optimizing the detection direction of the photoelectrons, but it sometimes makes it difficult to normalize spectra recorded at different angles. This might be simply due to the surface roughness; the area of the excitation X-ray on a rough surface is often not exactly the same when the sample surface is not flat, yielding a variation of the photoemission intensity. In the case of a very weak dichroic signal, as shown in Fig. 4 ▸, it is crucial to record the photoemission signal from the same region on the sample surface for comparison of spectra before and after any rotations. To minimize this problem, we have also installed a co-axial sample monitoring system (Fig. 5 ▸) combining a long-working-distance optical microscope (Infinity photo-optical, K2/SC) and an aluminium mirror (50 mm square) having a 5 mm-diameter through-hole for the X-ray beam as shown in Fig. 5(*b*) ▸.

**Figure 6:**
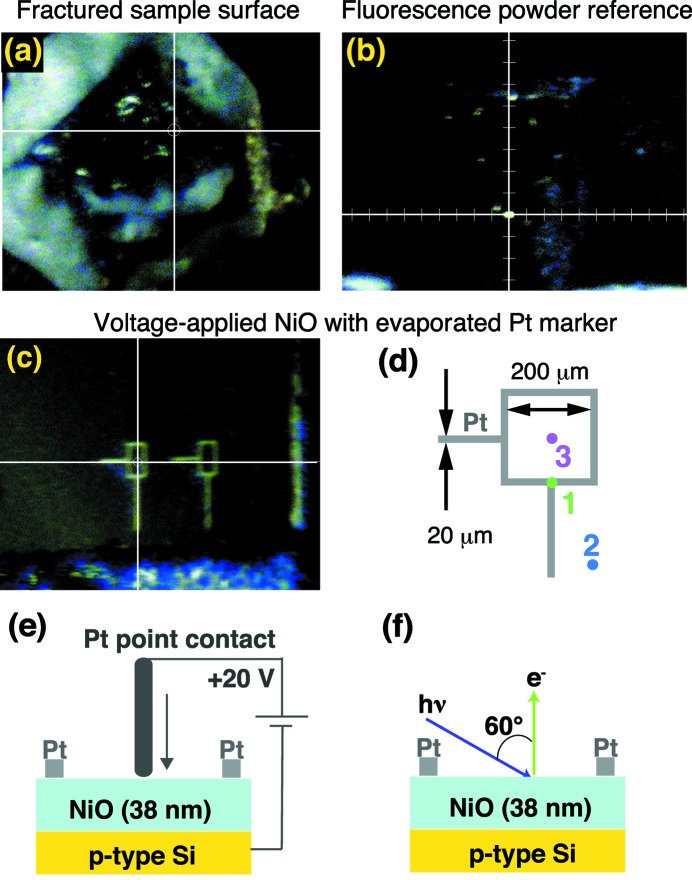
Co-axial microscope image of the fractured surface of YbB_12_ (*a*), of the fluorescence-powder reference on the gold reference on the manipulator (*b*), and of the evaporated Pt marker on NiO film (*c*). The X-ray beam spot is located at the intersection of the cross-lines. (*d*) Schematic image of the square- and line-shaped Pt marker for defining specific regions on the NiO. The positions measured by HAXPES are also indicated on the Pt marker (1), as-grown NiO film (2) and the voltage-applied region on NiO (3). (*e*) Schematic view of the setup for applying the voltage on the NiO film by Pt point contact outside the photoemission chamber, and (*f*) the experimental geometry of the HAXPES measurement.

As can be seen in Fig. 6(*a*) ▸, the sample position is selected using a micro-positioning technique with similar monitoring systems (Muro *et al.*, 2009 ▸, 2011 ▸; Fujiwara *et al.*, 2015 ▸). The intersection between the incoming X-ray beam and the photoelectron analyzer axis is marked beforehand onto the microscope monitor using a fluorescent substrate, which is positioned to maximize the counts of photoelectrons detected by the photoelectron analyzer. To adjust a certain region on the sample surface to this intersection, we first set the target region to the mark on the microscope monitor, and scanned the sample position along the beam axis while keeping the target region on the mark until detecting the maximum photoelectron-counts to avoid the ambiguity of the focal depth of the microscope. Note that we have a gold reference with a small amount of phosphor powder on the aluminium finger as shown in Fig. 3(*b*) ▸ to mark the beam spot any time as in Fig. 6(*b*) ▸. There is no serious problem caused by the phosphor powder on the gold surface for optimizing the count rate owing to the high photoelectron kinetic energy and long probing depth at HAXPES. This simple device is helpful if one changes the magnification of the microscope by changing the lens system of the microscope. The positioning error is within ±50 µm evaluated with the patterned Pt markers evaporated on NiO as shown in Figs. 6(*c*) and 6(*d*) ▸. This error is mainly due to vibration of the closed-cycle refrigerator.

By utilizing this positioning system we have studied the electronic structure of NiO, which is a benchmark of strongly correlated insulators (Sawatzky & Allen, 1984 ▸; Zaanen *et al.*, 1985 ▸), for application as non-volatile resistive random access memory (Gibbons & Beadle, 1964 ▸; Kim *et al.*, 2006 ▸; Calka *et al.*, 2011 ▸; Jeong *et al.*, 2012 ▸; Horiba *et al.*, 2013 ▸). We applied a voltage to the NiO film by the Pt point contact for switching the resistivity at 50 places in a region of 200 µm × 200 µm, indicated by the square-shaped Pt marker on the NiO as shown in Figs. 6(*c*) and 6(*d*) ▸. We set the metallic states on NiO by sweeping the voltage from 0 V to +20 V between the Pt point contact touching on the NiO film and the p-type Si substrate, as shown in Fig. 6(*e*) ▸, then release the voltage to 0 V while keeping the metallic conductivity due to the non-volatility. For studying the electronic structures of this sample by HAXPES in a different chamber using synchrotron radiation, we have chosen three measurement positions: the Pt marker (position 1), the as-grown NiO film (position 2) and the voltage-applied NiO (position 3), indicated in Fig. 6(*d*) ▸. The closed-cycle He refrigerator was switched off to minimize the vibration of the manipulator with the positioning error to ±25 µm. Thus the measured temperature was set to 300 K and the overall energy resolution was set to 400 meV with the normal emission geometry as shown in Fig. 6(*f*) ▸.

**Figure 7:**
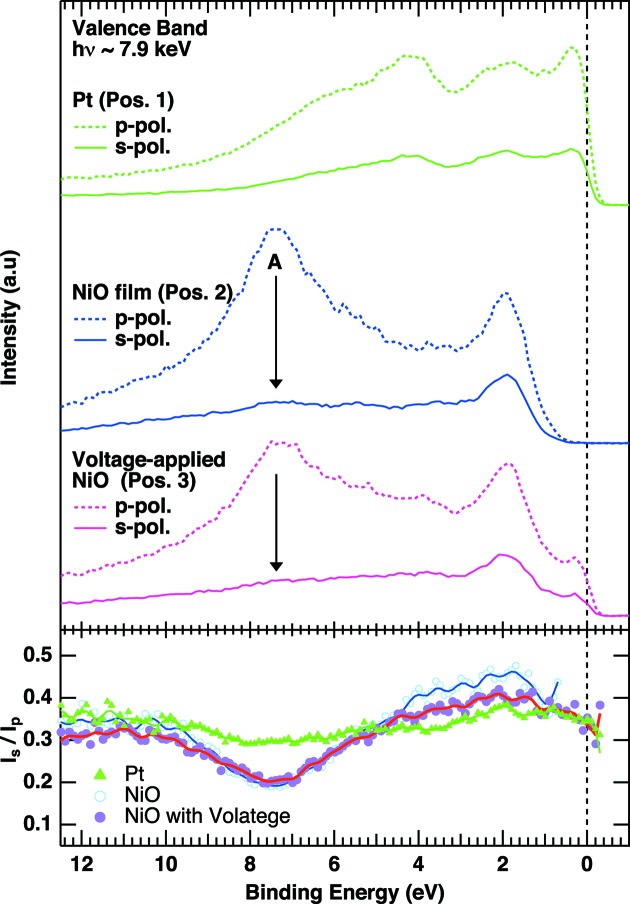
Valence-band photoemission spectra of Pt measured at position 1 (Pos. 1) in Fig. 6(*d*) ▸, those of the as-grown NiO substrate (Pos. 2) and those of the voltage-applied NiO (Pos. 3) (bottom) recorded by the *p*- and *s*-polarized photons. 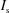/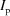 spectra for all three positions are plotted in the lower panel. Note that the data points ranging from 0.4 eV to the Fermi level in the 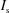/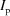 spectrum for as-grown NiO are removed to eliminate the divergent behavior due to the gap opening.

Fig. 7 ▸ shows polarization-dependent valence-band HAXPES spectra recorded at these three positions. The valence-band spectra recorded on the Pt marker, namely at position 1, show the clear Fermi cut-off, and the spectral shape is not very sensitive to the polarization (Yamasaki *et al.*, 2014 ▸). Meanwhile, the spectra for as-grown NiO at position 2 outside the Pt marker clearly show the gap opening near the Fermi level. Here clear polarization dependence of the line shape is observed; namely, the Ni 4*s* contribution is much enhanced in the *p*-polarized geometry, especially at the position of peak A around 7.5 eV (Sekiyama *et al.*, 2010 ▸; Nakatsu *et al.*, 2011 ▸; Weinen *et al.*, 2015 ▸). This is due to the asymmetry factors of the photoionization cross sections (Trzhaskovskaya *et al.*, 2001 ▸, 2002 ▸, 2006 ▸). We note that the spectral line shape of the valence-band spectra of the as-grown NiO is essentially the same as that reported by Weinen *et al.* (2015 ▸), in which the valence-band spectra of NiO were recorded in the complete *s*- and *p*-polarized geometries by changing the analyzer position. Therefore we stress that our polarization switching system works successfully with a high degree of linear polarization on the sample position.

**Figure 8:**
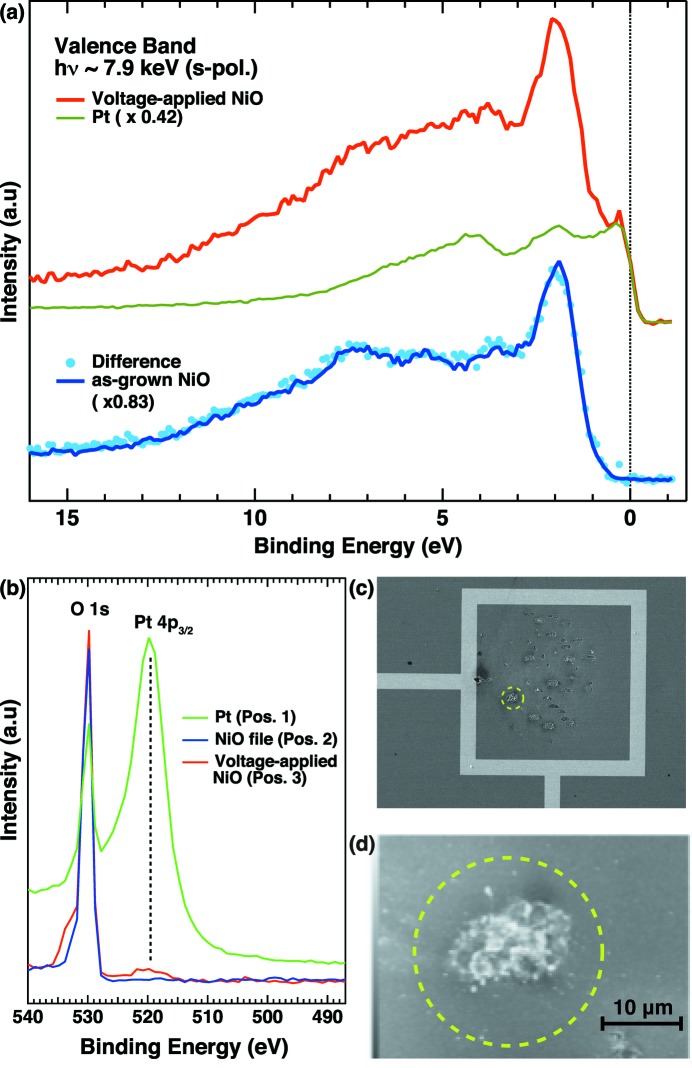
(*a*) Valence-band photoemission spectrum with *s*-polarization geometry for the voltage-applied NiO and normalized spectrum of Pt (top). The difference is shown at the bottom with the spectrum of the as-grown NiO film. (*b*) Pt 4*p*_3/2_ core-level photoemission spectra. (*c*) SEM image for the voltage-applied NiO. The dashed circle indicates one of the spots touched by the Pt point contact, with a focused SEM image shown in (*d*).

Compared with the as-grown NiO, the metallic Fermi cut-off is clearly observed at position 3 in the voltage-applied NiO in Fig. 6(*d*) ▸. To identify the origin of the metallic cut-off, we have compared the intensity ratio of the *s*-polarized (*p*-polarized) spectra (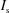/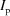) for all three positions in the lower panel of Fig. 7 ▸, for resolving the orbital contributions in the valence-band spectra (Sekiyama *et al.*, 2010 ▸). 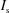/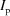 for the voltage-applied NiO overlaps the as-grown spectra in the range from 12 eV to 5 eV, suggesting the valence-band character is not heavily modified in this binding energy range after application of the voltage. Meanwhile, 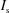/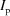 for the voltage-applied NiO ranging from 5 eV to 1 eV is somewhat in between those of the Pt and as-grown NiO, and overlaps fully with that of Pt near the Fermi level, suggesting a contribution from Pt in this region on the voltage-applied NiO. We have further compared the spectral line shape of voltage-applied NiO with the Pt valence-band spectra in Fig. 8(*a*) ▸. The normalized Pt valence-band spectrum, so as to fit the slope of the metallic Fermi cut-off, completely overlaps with the spectrum of the voltage-applied NiO near the Fermi level. The subtracted spectrum of the normalized Pt spectrum from the raw spectrum of the voltage-applied NiO quantitatively reproduces the spectrum of the as-grown NiO. The Pt contribution in the voltage-applied NiO is also supported by a weak but finite spectral weight due to the Pt 4*p*_3/2_ core-level contributions in Fig. 8(*b*) ▸. We should note that the Pt contribution is not due to the surrounded Pt because the length of the inner side of the marker is 200 µm while the footprint of the X-ray beam on the sample surface is 25 µm × 50 µm (FWHM) for the normal emission geometry with grazing angle of 30° in which the sample normal is inclined by 60° to the X-ray beam as shown in Fig. 6(*f*) ▸. Since the spatial distribution of the photoelectrons recorded by the photoelectron spectrometer was well focused, the signal from the surrounded Pt can be excluded from the spectral shape of the voltage-applied NiO.

Indeed, the scanning tunneling microscope (SEM) image for the region of voltage-applied NiO shows many imprints formed by applying the voltage with Pt point contact as shown in Fig. 8(*c*) ▸. In particular, the focused SEM image (Fig. 8*d* ▸) for one of the imprints indicated by the dashed circle in Fig. 8(*c*) ▸ clearly shows a bump-like structure, where the energy-dispersive X-ray image (not shown) revealed the fluorescence signals from the Pt *M*-edge. Therefore, the origin of the metallic cut-off could be due to diffused Pt from the point contact during application of the voltages for switching the resistivity. This provides important feedback for improving the method of preparation of the electrical contact for studying the electronic structure of NiO-based resistive random access memory.

## 3. Conclusion   

We have developed a HAXPES system equipped with a high-throughput X-ray phase retarder, a low-temperature two-axis manipulator and a co-axial microscope for revealing the ground-state symmetry of strongly correlated electron systems. The two-axis manipulator is capable of rotating by 90° for azimuthal and polar angles with a lowest cooling temperature achieved of 9 K by closed-cycle He refrigerator, which gives a better chance to study the electronic structure of the ground states in various rare-earth compounds. The co-axial sample monitoring system with a long-working-distance optical microscope supports recording of the reliable polarized HAXPES spectra by keeping the same sample position before and after rotation. By utilizing this micro-positioning system, we have demonstrated linear polarization-dependent valence-band HAXPES for voltage-applied NiO in order to study the resistive switching phenomena and found that Pt could be diffused on NiO when a voltage was applied, forming metallic states near the Fermi level.
